# Targeting phospholipase PLAG-15 promotes healthy aging in *C. elegans* via lysosomal-related genes

**DOI:** 10.1016/j.isci.2025.112880

**Published:** 2025-06-16

**Authors:** Sanne van der Rijt, Marte Molenaars, Rashmi Kamble, Weisha Li, Bauke V. Schomakers, Adrie D. Dane, Simone W. Denis, Michel van Weeghel, Frédéric M. Vaz, Alessandra Tammaro, Georges E. Janssens, Arwen W. Gao, Riekelt H. Houtkooper

**Affiliations:** 1 Laboratory Genetic Metabolic Diseases, Department of Laboratory Medicine, Amsterdam UMC Location University of Amsterdam, Meibergdreef 9, Amsterdam, the Netherlands; 2 Pathology Research Laboratory, Amsterdam UMC Location University of Amsterdam, Meibergdreef 9, Amsterdam, the Netherlands; 3 Amsterdam Gastroenterology, Endocrinology, and Metabolism, Amsterdam, the Netherlands; 4 Core Facility Metabolomics, Amsterdam UMC Location University of Amsterdam, Meibergdreef 9, Amsterdam, the Netherlands; 5 Department of Clinical Epidemiology, Biostatistics, and Bioinformatics, Amsterdam UMC Location University of Amsterdam, Meibergdreef 9, Amsterdam, the Netherlands; 6 Amsterdam Cardiovascular Sciences, Amsterdam, the Netherlands

**Keywords:** Molecular physiology, Lipidomics, Transcriptomics

## Abstract

Complex lipid metabolism plays a crucial role in regulating aging. We recently discovered that the phospholipid bis(monoacylglycero)phosphate (BMP) increases in aged human muscles and many mouse tissues. The phospholipase PLA2G15 is reportedly involved in BMP synthesis, however, its specific role in aging remains unknown. To elucidate the role of PLA2G15 in aging, we used *Caenorhabditis elegans* as a model. When silencing *plag-15*, the predicted worm orthologue of *PLA2G15,* we observed improved healthspan and lifespan extension. Semi-targeted lipidomics highlighted that instead of changes related to BMP, *plag-15* RNAi led to lower levels of lysophosphatidic acid, lysophosphatidylcholine, and lysophosphatidylethanolamine. Transcriptome-guided epistasis experiments identified that the lifespan extension of *plag-15* RNAi worms is regulated by transcription factors *hlh-30* and *elt-3*, and lysosomal vitamin B12 transporter *pmp-5* (human *TFEB*, *GATA*, and *ABCD4* respectively). Overall, we conclude that targeting phospholipid remodeling through *plag-15* could be a promising strategy to promote healthy aging.

## Highlights

• *plag-15* RNAi extends both healthspan and lifespan in *C. elegans*
• *plag-15* RNAi lowers LPA, LPC, and LPE lysophospholipid levels
• Lifespan extension mediated by *plag-15* RNAi requires *pmp-5*, *hlh-30*, and *elt-3*

## Graphical abstract

**Figure:**
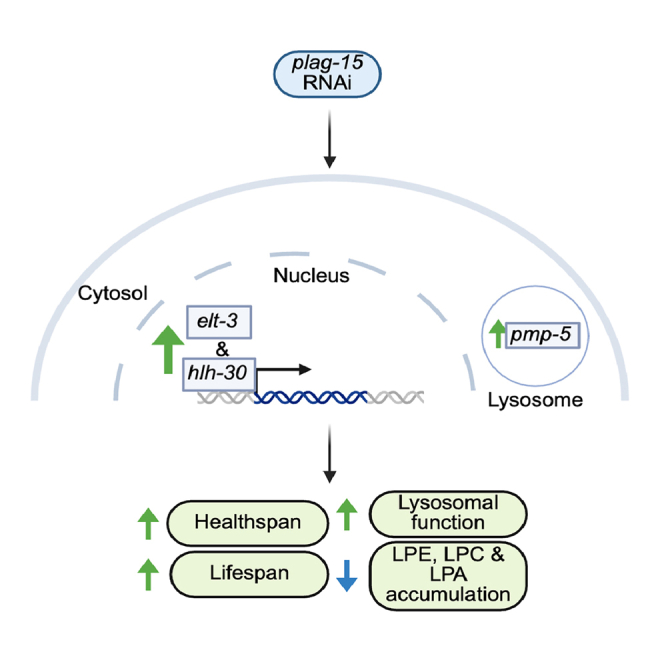

## Introduction

Aging is one of the main risk factors for age-related diseases, such as cardiovascular disease, cancer, and neurodegenerative diseases. Genetic and pharmacological interventions can reduce the burden of age-related diseases and promote healthy aging by acting on one or multiple mechanistic hallmarks of aging.^1^^,^^2^ Several hallmarks of aging have been classified, including deregulated nutrient sensing, mitochondrial dysfunction, cellular senescence, and altered cellular communication.^3^^,^^4^

One of the key overarching mechanisms driving aging hallmarks relates to metabolic disturbance. Indeed, metabolic changes are consistently linked to aging hallmarks, and these factors are known to functionally affect each other.^5^ An emerging theme in relation to metabolic changes involves complex lipids.^6^ Complex lipids are defined as lipids with three or more chemical moieties (e.g., glycerol, phosphate, and fatty acids). Lipids are structural components of the cell membrane and important for energy storage and signaling pathways.^7^ In several diseases, changes in complex lipid metabolism have been described. In Barth syndrome, an inherited metabolic disorder, cardiolipin remodeling is defective and the abundance of cardiolipin is reduced while the metabolic intermediate monolysocardiolipin accumulates.^8^ Furthermore, sphingolipids accumulate in aging mice and preventing this accumulation via genetic and pharmacological interventions leads to, for instance, enhanced strength.^9^ Besides this, complex lipid composition changes in kidneys of mice with high fat diet–induced chronic kidney disease, including increased abundance of bis(monoacylglycero)phosphate (BMP),^10^ a lipid class that is located in the (endo)lysosomal compartment and is critical for lysosomal and cellular integrity and function.^11^ We recently showed that in several tissues in mice, including the kidney, liver, and muscle, BMP accumulates with age.^12^ Furthermore, BMP accumulates in muscles from older human individuals. Conversely, an intervention to promote metabolic health, consisting of exercise, lowered the BMP levels.^12^

Phosphatidylglycerol (PG) is the precursor of BMP. Although the pathway for BMP synthesis has long remained obscure, it has been reported that the phospholipase A2, *PLA2G15*, converts PG into lysophosphatidylglycerol (LPG) *in vitro*, which is a precursor to BMP.^13^,^14^ Next, LPG is converted into BMP by CLN5.^15^ *PLA2G15* is a late endosomal/lysosomal enzyme phospholipase capable of hydrolyzing short-chain (lysosomal) phospholipids.^14^ However, not much is known about the exact role of *PLA2G15* in BMP synthesis and aging. Therefore, we aimed to investigate whether targeting *PLA2G15* could slow down the aging process and lead to improved healthspan and extended lifespan through changes in BMP.

To test this hypothesis, we used the nematode *Caenorhabditis elegans*, a widely employed model for studying aging and longevity.^16^ Searching for the *C. elegans* orthologue of *PLA2G15* by using Ensembl, we found that *plag-15* is the predicted orthologue of *PLA2G15.* Unfortunately, the *C. elegans* orthologue of *CLN5* is not known. Interestingly, *plag-15* RNAi resulted in longevity and increased healthspan in worms. Strikingly, comprehensive lipidomics of *plag-15* RNAi worms revealed no changes in PG, LPG, or BMP. Instead, we observed alterations in lysophosphatidic acid (LPA), lysophosphatidylcholine (LPC), and lysophosphatidylethanolamine (LPE). These findings suggest that PLAG-15 may influence worm lifespan through other phospholipid pathways rather than through BMP. Based on RNA-seq analysis and the subsequent epistasis experiments, we found that transcription factors *hlh-30/TFEB* and *elt-3*/*GATA* were required for the lifespan extension of *plag-15* RNAi. Additionally, the lysosomal vitamin B12 transporter *pmp-5* (*ABCD4* in mammals) was required for this lifespan extension. Altogether, our results suggest that modulating phospholipid remodeling via *plag-15* represents a therapeutic strategy to promote healthy aging.

## Results

### Knockdown of lysosomal enzyme *plag-15* prolongs lifespan and healthspan in *C. elegans*

**Figure 1:**
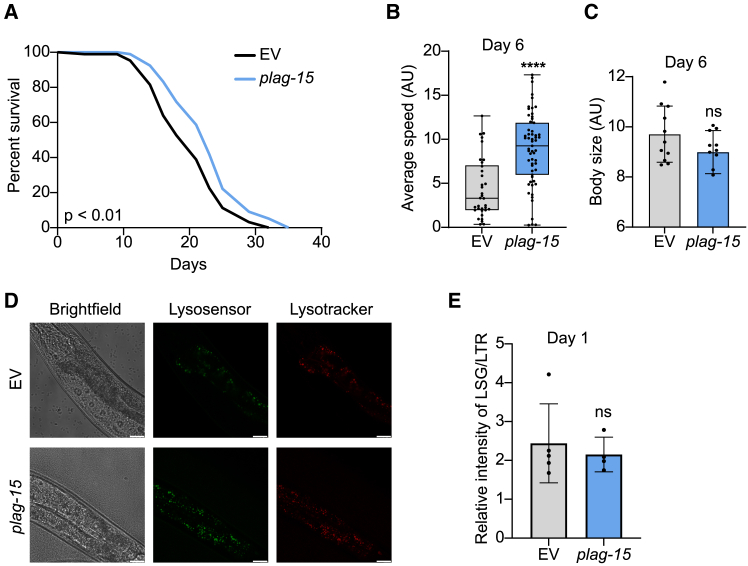
Knockdown of *plag-15* results in increased lifespan and healthspan, without changes in lysosomes (A) *plag-15* RNAi worms have an increased lifespan. *plag-15* RNAi consists of 50% *plag-15* RNAi and 50% empty vector (EV) control RNAi. Statistical analysis was performed by log rank test, see Table S1 for lifespan statistics. (B) Knockdown of *plag-15* (*n* = 59) promotes the mobility of the day 6 adult worms compared to the control worms (*n* = 34). Statistical analysis was performed by t-test, error bars denote the quartiles. (C) Body size of *plag-15* in arbitrary units (AU). No differences were found in worm size between EV (*n* = 11) and *plag-15* RNAi (*n* = 10) worms. Statistical analysis was performed by t-test, error bars denote standard deviation. (D) Confocal images of *plag-15* knockdown worms and control. Lysosensor is shown in green and lysotracker in red. No differences were found in lysosomal mass. Scale bar = 40 μm. (E) Quantification of lysosomal imaging showed no differences in lysosomal mass in control (*n* = 5) versus *plag-15* (*n* = 4). See Table S1 for lifespan statistics. ∗∗∗∗*p* < 0.0001. Data are presented as mean ± SD.

Building on our previous finding that BMP levels increase in aged mouse tissues and aged human muscles, we aimed to decipher the causal role of *PLA2G15* in aging by using the nematode *C. elegans* as a model. We first conducted a homology search via Ensembl for *PLA2G15* and found that *plag-15* was its predicted *C. elegans* orthologue. Knocking down the expression of *plag-15* in worms using RNAi led to a consistent median lifespan extension (*p* < 0.01), suggesting that *PLA2G15* plays a regulatory role in aging (Figure 1A), in line with our BMP findings.^12^ Next, we measured the healthspan in *C. elegans* by analyzing their moving speed for 20 s.^17^ We observed an improved healthspan in *plag-15* RNAi worms compared to the controls at day 6 of adulthood, indicating an overall improvement in health (Figure 1B). The body size of *plag-15* RNAi worms was similar to the control worms (Figure 1C), suggesting that *plag-15* RNAi prolongs worm lifespan without affecting morphology.

Given that *plag-15* encodes a protein that functions in the lysosome, we examined the effects of *plag-15* RNAi on lysosomes. To investigate the lysosomal activity, we used the fluorescent intensity ratio of LysoSensor Green (LSG) to LysoTracker Red (LTR). LSG visualizes the lysosomal acidity, important for the function of lysosomes, and a parameter that declines with age.^18^ LTR is used to normalize for the dye intake of *C. elegans*.^19^ We observed no significant differences in lysosomal acidity in *plag-15* RNAi compared to the controls (Figures 1D and 1E). Collectively, these results suggest that knocking down *plag-15* promotes healthy aging without influencing the body size and lysosome activity in *C. elegans*.

### Lipidome of *plag-15* RNAi worms shows lowered lysophospholipid levels

**Figure 2:**
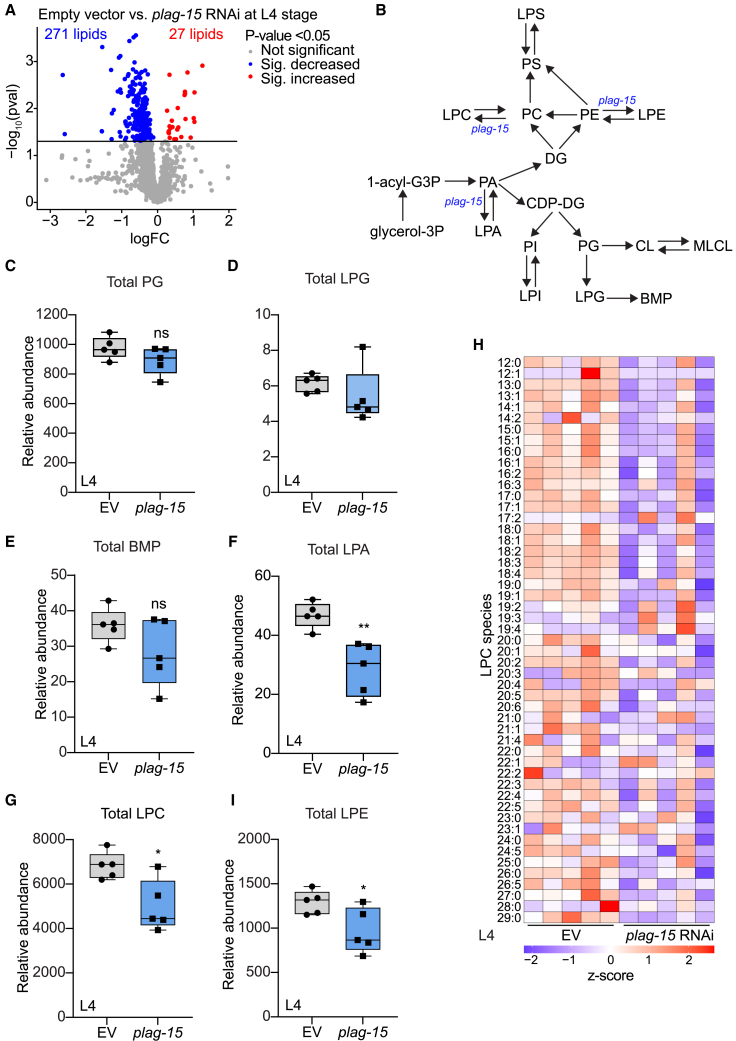
Lipidome of *plag-15* RNAi worms show decreased lysophosphatidyl acid (LPA) lysophosphatidylcholine (LPC) and lysophosphatidylethanolamine (LPE) compared to control worms at the L4 stage (A) Volcano plot of L4 worms, *plag-15* versus empty vector (EV). Blue is the lipids that are decreased in abundance in *plag-15* RNAi (271 lipid species), while red are the lipids that are increased upon *plag-15* RNAi (27 lipid species). (threshold: *p*-value <0.05). (B) The schematic overview of the phospholipid pathway. Glycerol-3P: Glycerol 3-phosphate; 1-acyl-G3P: 1-acylglycerol-phosphate; DG: diacylglycerol; CDP-DG: cytidine diphosphate diacylglycerol; PS: Phosphatidylserine; LPS: lysophosphatidylserine; PC: phosphatidylcholine; LPA: lysophosphatidic acid; LPC: lysophosphatidylcholine; PE: phosphatidylethanolamine; LPE: lysophosphatidylethanolamine; PA: phosphatidic acid; PI: phosphatidylinositol; LPI: lysophosphatidylinositol; PG; phosphatidylglycerol: CL; cardiolipin: MLCL; monolysocardiolipin: LPG; lysophosphatidylglycerol: BMP; bis(monoacylglycero)phosphate. (C–I) Total PGs (C), total LPGs (D), total BMPs (E), total LPAs (F), total LPCs (G), heatmap of LPC species (H), total LPE (I) in L4 *plag-15* RNAi worms compared to EV worms. Statistical comparison determined by using the unpaired t-test was ∗: *p* < 0.05; ∗∗: *p* < 0.01. *N* = 5 for each group. Data are presented as mean ± SD.

As *plag-15* functions as a phospholipase in the lysosome, and is expected to act on BMP levels,^14^ we examined the lipidome of *plag-15* RNAi worms at both the larval stage 4 (L4) and day 6 of adulthood (Table S3). At L4 stage, we identified 27 increased lipid species and 271 decreased lipid species in *plag-15* RNAi worms compared to empty vector controls, respectively (Figure 2A). Principal component analysis showed a clear separation between our control and the *plag-15* RNAi worms (Figure S1A). Furthermore, the heatmap of all the lipid species show lower abundance of lysophospholipids in *plag-15* RNAi worms compared to control (Figure S1B.) In contrast to our expectations, BMP synthesis intermediates PG and LPG and BMP itself was not changed in L4 worms (Figures 2B–2E), and the same is true at day 6 (Figures S2A–S2D). Since there were no changes in the pathway of BMP synthesis, we started to investigate other changes. Mainly lysophospholipids were significantly altered upon *plag-*15 RNAi, especially at the L4 stage. Indeed, both the total class and the individual species of lysophosphatidic acid (LPA, Figure 2F; Figure S2E) were significantly lowered in L4 worms. Similarly, many LPC species, including one of the most abundant LPC lipid species,^20^ LPC18:0, were significantly decreased (Figures 2G and 2H; Figure S2F). As were the total class of lysophosphatidylethanolamine (LPE) levels and many of the individual LPE species (Figures 2I; Figures S2G–S2H). However, the precursors of LPA, LPC, and LPE (i.e., PA, PC, and PE) were not significantly changed upon *plag-15* RNAi (Figures S2I–S2K). Our data suggests that knockdown of *plag-15* does not influence BMP synthesis. However, LPA, LPC, and LPE are significantly decreased in *plag-15* RNAi worms.

### Transcripts involved in defense response and lysosomes are upregulated in *plag-15* knockdown worms

**Figure 3:**
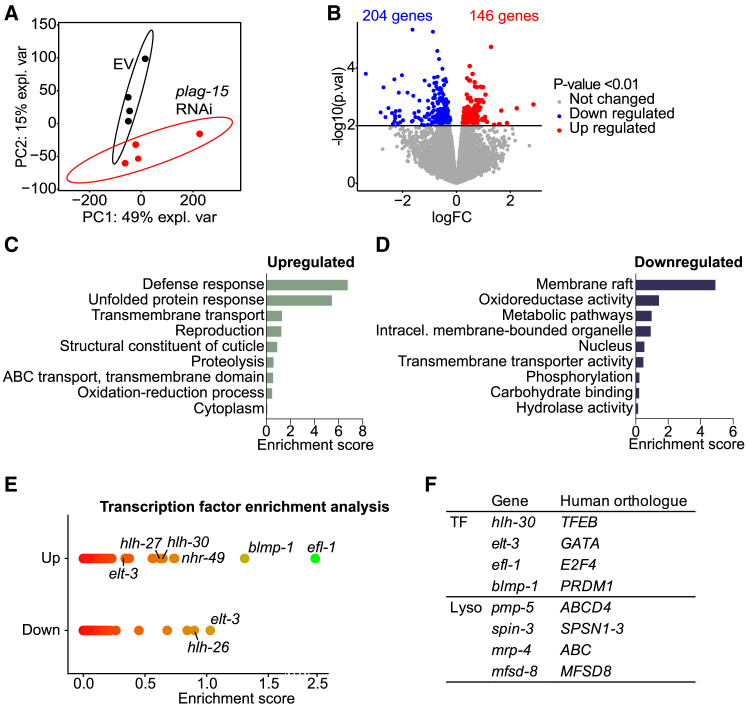
RNA-seq of *plag-15* knockdown worms showed an increased defense response and upregulation of lysosomal genes (A) Principal Component Analysis (PCA) plot of the transcriptome showing group separation based on the lipidome in *plag-15* RNAi versus empty vector (EV) control. (B) Volcano plot of the RNAseq results, (threshold non-adjusted *p*-value: *p* < 0.01). (C and D) Pathway enrichment analysis of upregulated (C) and downregulated (D) transcripts (non-adjusted *p*-value: *p* < 0.01) in *plag-15* RNAi worms compared to the controls. (E) Transcription factor enrichment analysis of the up-/down-regulated transcripts. (F) Table of candidate genes including the transcription factors (TFs) that show increased transcription and lysosomal (lyso) genes that are upregulated in *plag-15* RNAi worms. *N* = 4 for each group. Data are presented as mean ± SD.

To untangle how *plag-15* plays a role in longevity, we performed RNA sequencing (RNA-seq) on total RNA isolated from L4 worms (Table S4). PCA showed a clear separation between the control and *plag-15* RNAi worms (Figure 3A). 146 genes were upregulated and 204 genes were downregulated in *plag-15* RNAi worms, compared to the control (Figure 3B). We then performed gene ontology (GO) term enrichment analysis to investigate which biological pathways were altered in *plag-15* RNAi worms. Defense response pathways and the unfolded protein response (UPR) were significantly upregulated in *plag-15* RNAi worms (Figure 3C), these pathways that are often associated with longevity and lipid metabolism is known to play a role in regulating both defense response and the UPR.^21^ However, the role of these pathways in *plag-15* and phospholipid metabolism remain poorly understood. In addition, GO terms related to structural constituent of the cuticle, transmembrane transport, and reproduction were also upregulated (Figure 3D). Given the significant upregulation of defense response and UPR, we investigated the involvement of genes within these GO terms in lifespan regulation of *plag-15* RNAi (Figures S3A–S3C). We found that *vit-5* (defense response) and *abu-8* (UPR) do not contribute to *plag-15-*mediated longevity. Notably, the knockdown of *vit-6*, a defense response gene, attenuated the lifespan extension induced by *plag-15* RNAi, suggesting that *vit-6* plays a critical role in mediating the longevity effects of *plag-15* RNAi (Figures S3A–S3C). When looking at the downregulated GO terms, we found genes mainly associated with the membrane rafts, oxidoreductase activity, and metabolic pathways (Figure 3D). Furthermore, genes related to intracellular membrane-bound organelles, nucleus, transmembrane transporter activity, phosphorylation, carbohydrate binding, and hydrolase activity were downregulated (Figure 3D).

Next, to identify the key drivers of the downstream longevity responses in *plag-15* RNAi, we performed transcription factor enrichment analysis with the transcript profile of *plag-15* RNAi via Worm EnrichR (Figure 3E).^22^^,^^23^The transcription factors that were enriched in *plag-15* RNAi treated worms included *elf-1*, *blmp-1*, *nhr-49*, *hlh-30*, *hlh-27*, and *elt-3* (Figure 3E). The human orthologue of *efl-1* is E2F, a transcription factor that is involved in cell proliferation.^24^ Furthermore, *blmp-1* regulates the timing of larval development of the worm and regulates programmed cell death.^25^ *Nhr-49* is involved in regulating the fatty acid composition of *C. elegans*, and regulates the breakdown of lipids.^26^ The transcription factor *hlh-30* is the orthologue of human *TFEB*, the master regulator of lysosomal biogenesis and autophagy, and knocking down *hlh-30* results in a shorter lifespan in worms.^27^ *Hlh-27* is part of the *ref-1* family, involved in Notch signaling in *C. elegans*, and is enriched in the upregulated genes, whereas *hlh-26* is enriched in the downregulated genes.^28^ Lastly, *elt-3* is a GATA transcription factor, also involved in lysosomal function and the development of *C. elegans*, which was enriched in both the up- and downregulated genes.^29^ From the transcription factors that were enriched in the upregulated genes, we made a candidate list for follow-up. Since *plag-15* is a lysosomal enzyme, we prioritized genes and transcription factors that are involved in lysosomal function, such as *hlh-30* and *elt-3*. Many genes that are regulated by *elt-3* (e.g., *pud-3, thn-2, pitp-1, T06D8.9, nos-1*, *and folt-2*) and *hlh-30 (*e.g., *C02B10.6, hsp-43, ttbk-2, T22B7.7, cysl-1*, *and oma-1*) were increased in the RNA-seq dataset (Figures S4A and S4B). Furthermore, we selected the transcription factor which was most prominently enriched, *efl-1* (Figure 3F).

In addition to transcription factors, we performed a candidate gene approach for regulators of the *plag-15* longevity effect. As *plag-15* is a lysosomal enzyme, lysosomal function and signaling may also play an essential role in mediating the longevity effect induced by *plag-15* RNAi. Therefore, we specifically looked into the RNA-seq data to investigate which lysosomal genes are upregulated in *plag-15* RNAi worms. First, *pmp-*5 gene expression is upregulated in *plag-15* RNAi worms. *Pmp-5* is the orthologue of human *ABCD4*, the lysosomal vitamin B12 transporter. Vitamin B12 is essential for several metabolic pathways, such as the S-adenosylmethionine and S-adenosylhomocysteine (SAM/SAH) and in the breakdown of branched-chain amino acids.^30^^,^^31^ A deficiency in vitamin B12 can increase the risk of age-related diseases such as cardiovascular disease, cognitive dysfunction, and dementia.^32^ Additionally, *spin-*3 expression was increased in *plag-15* RNAi worms. *Spin-3* is the worm orthologue of human *SPNS-1*, the lysosomal transporter of the phospholipids LPC and LPE.^33^ Another lysosomal transporter gene that was increased in *plag-15* RNAi is *mrp-4*. *Mrp-4* is the human ATP binding cassette transporter, important for lipid transport in the lysosomes.^34^ Finally, *mfsd-8* (human orthologue *CLN-7*) is a lysosomal chloride channel important for lysosomal function, and mutations of *CLN-7* lead to a lysosomal storage disorder.^35^^,^^36^ We selected these genes as candidates for the regulation of *plag-15* RNAi-mediated lifespan extension (Figure 3F).

### Transcription factors *hlh-30* and *elt-3* and lysosomal transporter *pmp-5* are required for *plag-15* RNAi-mediated lifespan extension

**Figure 4:**
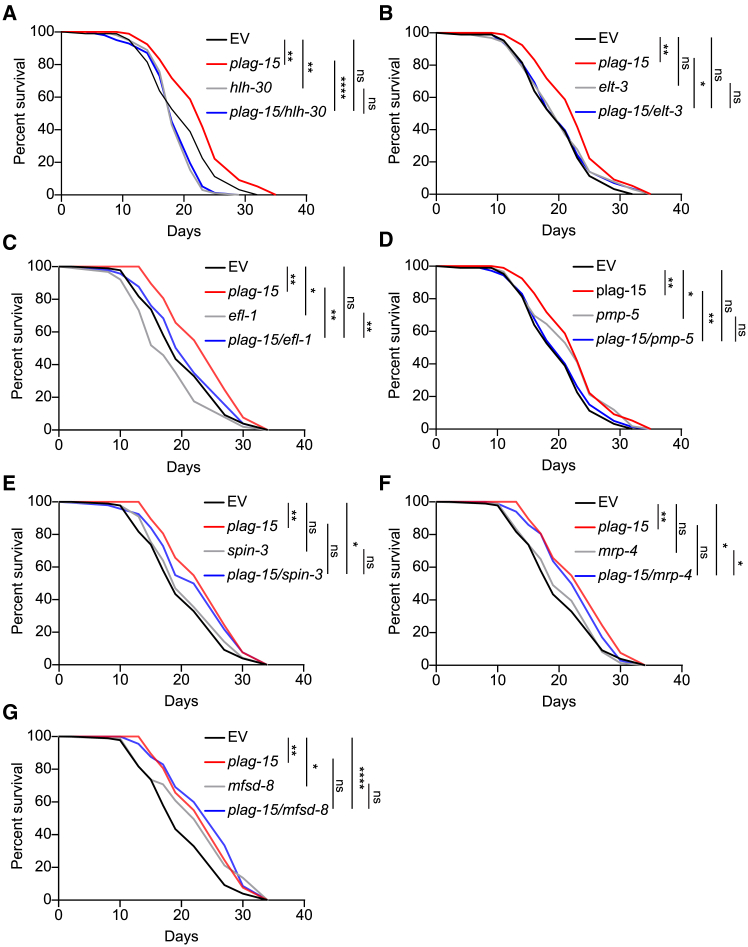
Lifespan screen of candidate genes from the RNAseq data showed that lifespan extension of *plag-15* RNAi is dependent on several genes (A–C) Lifespan curves of the transcription factor enrichment analysis from RNA-seq data of the *plag-15* RNAi worms. (A) Lifespan curve of EV, *plag-15*, *hlh-30* and *plag-15/hlh-30* RNAi. Lifespan extension of *plag-15* RNAi is dependent on *hlh-30.* (B) Lifespan curve of EV, *plag-15*, *elt-3* and *plag-15/elt-3* RNAi. Lifespan extension of *plag-15* RNAi is dependent on *elt-3.* (C) Lifespan curve of EV, *plag-15*, *efl-1* and *plag-15/efl-1. Efl-1/plag-15* RNAi still extends the lifespan compared to *efl-1* RNAi only. Therefore, *plag-15* RNAi is not dependent on transcription factor *efl-1*. (D–G) Lifespan curves of the lysosomal genes that emerged from the *plag-15* RNAi RNA-seq data. (D) Lifespan curve of EV, *plag-15*, *pmp-5* and *plag-15/pmp-5* RNAi. Lifespan extension of *plag-15* RNAi is dependent on *pmp-5.* (E) Lifespan curve of EV, *plag-15*, *spin-3* and *plag-15/spin-3* RNAi. *Spin-3/plag-15* RNAi still extends the lifespan compared to *spin-3* RNAi only. Therefore, *plag-15* RNAi is not dependent on transcription factor *spin-3.* (F) Lifespan curve of EV, *plag-15*, *mrp-4* and *plag-15/mrp-4. Mrp-4/plag-15* RNAi still extends the lifespan compared to *mrp-4* RNAi only. Therefore, *plag-15* RNAi is not dependent on transcription factor *mrp-4.* (G) Lifespan extension of *plag-15* is dependent on *mfsd-8. Mfsd-8/plag-15* RNAi still extends the lifespan compared to *mfsd-8* RNAi only. Therefore, *plag-15* RNAi is not dependent on transcription factor *mfsd-8.* Statistical comparison determined by using the log rank test was ∗*p* < 0.05 ∗∗*p* < 0.01, ∗∗∗*p* < 0.001, and ∗∗∗∗*p* < 0.0001. See Table S1 for lifespan statistics. Data are presented as mean ± SD.

In order to experimentally establish which mechanisms drive the lifespan extension of *plag-15* RNAi worms, we fed worms *plag-15* RNAi bacteria together with the RNAi bacteria targeting the candidate genes (Figure 3F), effectively creating a double knockdown scenario. First, we performed lifespan analysis on the three candidate transcription factors. The lifespan extension upon *plag-15* RNAi treatment was significantly attenuated when combined with either knockdown of *hlh-30* (Figure 4A) or *elt-3* (Figure 4B; Figure S4). Next, we took advantage of *hlh-30::gfp* reporter worms and performed confocal microscopy to assess HLH-30 translocation to the nucleus. Our results showed a significant increase in HLH-30 nuclear localization in *plag-15* RNAi worms compared to controls (Figure S5A). This observation suggests enhanced lysosomal signaling and autophagy in *plag-15* RNAi worms. We further assessed autophagy by measuring LGG-1 protein levels via Western blot and observed no significant changes in *plag-15* RNAi worms compared to the control (Figure S5B). This result suggests that *plag-15* RNAi likely mediates longevity through enhanced lysosomal function rather than through autophagy. Moreover, the lifespan extension of *plag-15* RNAi was not dependent on *efl-1* (Figure 4C). These data suggest that both *hlh-30* and *elt-3*, but not *efl-1*, are important for the lifespan extension of *plag-15* RNAi worms.

Next, we investigated the potential role of the four lysosomal candidate genes in the regulation of *plag-15* RNAi-mediated longevity. Knocking down the lysosomal vitamin B12 transporter *pmp-5* blunted the lifespan extension of *plag-15* RNAi (Figure 4D) without any changes in *plag-15* expression (Figure S6), while knockdown of *spin-3*, *mrp-4*, and *mfsd-8* did not have any effect on the longevity induced by *plag-15* RNAi (Figures 4E–4G).

Taken together, we conclude that the lifespan extension of *plag-15* RNAi is dependent on the lysosomal vitamin B12 transporter *pmp-5* as well as transcription factors involved in lysosomal biogenesis and activity *hlh-30* and *elt-3*.

## Discussion

**Figure 5:**
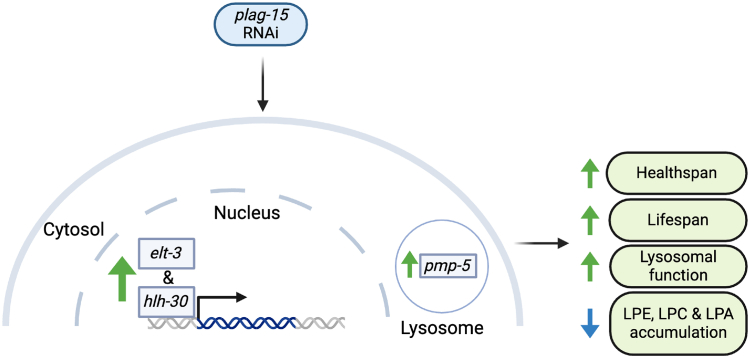
Mechanism of *plag-15* RNAi-mediated lifespan extension in *C. elegans* *Plag-15* RNAi leads to lifespan and healthspan extension in the worm *C. elegans*. We found that both transcription factors *elt-3* and *hlh-30* were necessary for the lifespan and healthspan extension in *plag-15* RNAi. Furthermore, lysosomal vitamin B12 transporter *pmp-5* was also required for the lifespan extension of *plag-15* RNAi. By performing lipidomics, we found a reduced abundance of the lysophospholipids lysophosphatidic acid (LPA), lysophosphatidylcholine (LPC) and lysophosphatidylethanolamine (LPE). Created with BioRender.com.

In this study, we aimed to explore how complex lipid metabolism influences aging, in light of our recent findings that aged mice exhibit high levels of BMP lipids in several organs compared to young mice and, likewise, older humans show elevated BMP lipids in muscles compared to young individuals.^12^ Based on these observations, we speculated that inhibiting BMP metabolism would lead to lifespan extension. By doing an orthologue search for *PLA2G15*, we found *plag-15* as the *C. elegans* orthologue for *PLA2G15.* Our experiments show indeed that *plag-15* RNAi prolongs lifespan and enhances healthspan in *C. elegans* (Figure 5).

While we originally focused on PLAG-15 due to its homology and possible roles in BMP synthesis, our lipidomic analyses did not reveal significant changes in BMP or its precursor LPG upon *plag-15* knockdown. Although several alternative explanations exist, it seems most likely that *plag-15* is not the functional homolog of *PLA2G15*, but instead encodes one of the other phospholipases of the PLA2 family. In line with this, we observed alterations in LPA, LPC, and LPE — lysophospholipid species that are increasingly recognized for their roles in membrane homeostasis and signaling pathways pertinent to aging. These findings suggest that PLAG-15 may influence worm lifespan through other phospholipid pathways rather than solely through BMP, and the phospholipase activity of *plag-15* may be involved in hydrolyzing PAs, PCs and PEs.^37^ However, the role of PLAG-15 in lysophospholipid regulation remains to be elucidated. By focusing on how PLAG-15 modulates LPA, LPC, and LPE, we sought to clarify its role in membrane homeostasis and aging beyond BMP-centric processes. LPA was previously linked to cancer and neurodegenerative disease through interaction with several G-protein coupled receptors, which are important for, for instance, cell proliferation.^38^ One of the receptors, *LPAR1*, shows increased activation in aged kidney, leading to inflammatory response and accelerated kidney aging.^39^ High levels of LPC can induce the production of reactive oxygen species, which can lead to accelerated aging.^5^^,^^40^ Furthermore, it has been shown that LPC is a pro-inflammatory lipid.^41^ Increased inflammation is one of the hallmarks of aging.^4^ Additionally, high levels of LPC can lead to fast progression of kidney dysfunction in patients with diabetic kidney disease leading to premature aging.^42^ Interestingly, our transcriptomics data showed increased expression of the lysosomal transporter *spin-3.* The human orthologue of *spin-3* is *SPSN-1*, which transports LPE and LPC out of the lysosome.^33^ Knockout of *Spsn1* in mouse liver led to accumulation of LPC and LPE and lysosomal dysfunction, resembling lysosomal storage disorders.^33^ Lowering LPC may be beneficial for cells, reducing the risk of ROS production, inflammation and maintaining lysosomal function. The phospholipases converting PA, PC, and PE into LPA, LPC, and LPE in *C. elegans* have not been characterized yet. Overexpression of these phospholipases would give us more insights in the mechanism of *plag-*15-mediated aging regulation and the role of the lysophospholipids in aging. Thus, our study represents an initial effort to delineate the mechanistic underpinnings of PLAG-15 in the regulation of lysophospholipid metabolism and its impact on healthy aging.

Our GO term analysis identified significant upregulation of genes involved in defense response, UPR, and transmembrane transport. Both defense response and UPR are well-documented pathways associated with increased lifespan.^21^^,^^43^ While lipid metabolism is known to play a role in regulating immune responses, the specific connections between *plag-15*, defense response, and UPR remain poorly understood and warrant further investigation. There is a strong correlation between lipid metabolism and these pathways. For instance, lipid metabolism is essential for the activation of immune responses in *C. elegans.*^43^ Additionally, several unfolded protein responses, such as the UPR^mt^, are closely linked to lipid metabolism. For instance, treating worms with doxycycline induces the UPR^mt^, which alters lipid metabolism and promote longevity.^44^^,^^45^ These findings highlight the intricate interplay between lipid metabolism, stress responses, and lifespan regulation, suggesting that *plag-15* may influence longevity through similar mechanisms.

Because *plag-15* is a lysosomal gene, we first sought to investigate whether its knockdown induces changes in lysosomal activity or integrity. Using LysoSensor and LysoTracker staining, we found no significant changes in lysosomal pH or quantity, suggesting that these parameters remain unaltered in *plag-15* RNAi worms. However, when we examined the potential involvement of transcription factors regulating lysosomal function, we observed a significant increase in HLH-30 translocation to the nucleus. This finding indicates an activation of the mild stress response, as HLH-30 is a key regulator of lysosomal biogenesis and function. The absence of changes in LysoSensor and LysoTracker staining, despite HLH-30 translocation, suggests that the mild stress response may be in its early stages or may involve pathways that do not directly alter lysosomal pH or quantity. Future investigation into downstream targets of HLH-30 and lysosomal functional assays will help clarify the mechanisms underlying this response.

Through analyzing the transcriptomics profile, we pinpointed the candidate genes that could explain the mechanism behind the lifespan extension induced by *plag-15* RNAi. By performing transcription factor enrichment analysis, we found *hlh-30* and *efl-1* as a candidate for *plag-15* RNAi lifespan extension and *hlh-30* was essential for the lifespan extension of *plag-15* RNAi. E2F, the mammalian orthologue of *efl-1*, is essential for cell cycle and cellular senescence.^46^ However, the role of E2F/*efl-1* in lipid and *plag-15* metabolism remains unknown. It has already been shown that *hlh-30* is important for lifespan extension and that it regulates lysosomal activity and autophagy^27^*.* TFEB, the mammalian orthologue of *hlh-30*, translocates to the nucleus upon starvation, where it upregulates genes important for lipophagy and lysosomal biogenesis. It is known that in mutant worms for *plag-15*, autophagy is impaired.^47^ Therefore, we hypothesized that *plag-15* is upregulating genes involved in autophagy via TFEB and therefore the transcription factor *hlh-30* is enriched in *plag-15* RNAi worms.^47^

Besides *hlh-30*, we found that *elt-3* is essential for the lifespan extension of *plag-15* RNAi worms. Another *elt* family gene, *elt-2*, regulates lifespan extension via the lysosomal surveillance response (LySR).^48^ Here, we found that the *elt-2* paralog *elt-3* is essential for the lifespan extension of worms treated with *plag-15* RNAi*.* It is known that *elt-3* is a GATA transcription factor in *C. elegans*, but its role in aging and lysophospholipid metabolism remains unknown.^49^^,^^50^ Finally, we demonstrated that *pmp-5*, and not *mrp-4*, is necessary for the lifespan extension of *plag-15* RNAi. MRP-4 is an endogenous and exogenous chemicals efflux transporter, however, not much is known about *mrp-4* and its role in complex lipid metabolism in aging.^51^ PMP-5 is the lysosomal vitamin B12 transporter. Vitamin B12 is critical to maintaining healthy lifespan,^52^ and is involved in several lipid-related metabolic pathways, for instance, in the S-adenosylmethionine and S-adenosylhomocysteine (SAM/SAH) pathway which is needed to provide one-carbon units for methylation reactions. Notably, the SAM/SAH pathway is critical for PC synthesis.^53^^,^^54^ This raises the possibility that the cells may upregulate vitamin B12 production to enhance LPC synthesis in the *plag-15* worms. However, the precise mechanism by which this pathway influences the lifespan of *plag-15* RNAi worms remains to be elucidated. Furthermore, the complex regulation of these target genes and their potential interplay through epistasis was not investigated in this manuscript and should be the scope of future research.

Altogether, our findings suggest that *plag-15* knockdown leads to lifespan extension in *C. elegans* via reducing abundance of various lysophospholipids and leading to increased transcription of *hlh-30* and *elt-3* and increased expression of the vitamin B12 transporter *pmp-5*. Based on this, we propose that targeting complex lipid metabolism might be a potential therapeutic target to promote healthy aging.

### Limitations of the study

Several limitations should be acknowledged in this study. While genes such as *pmp-5*, *mrp-4*, *spin-3*, *mfsd-8*, *efl-1*, *elt-3*, and *hlh-30* were identified as potentially relevant, their individual roles and potential interactions through epistatic mechanisms were not investigated. Future studies should explore interactions among these genes in greater depth. The causal link between *plag-15* and the observed reduction in lysophospholipids, including LPA, LPC, and LPE also remains to be elucidated. Although *plag-15* knockdown extended lifespan in *C. elegans*, the molecular mechanisms underlying this longevity effect are not yet fully understood. Furthermore, BMPs have emerged as intriguing lipid targets in the context of aging. However, the genes responsible for BMP synthesis and degradation in *C. elegans* have not yet been identified, and future work is required to elucidate the regulatory pathways involved. Finally, the regulatory relationship between the transcription factors HLH-30 and ELT-3 was not addressed. Whether these factors act independently or coordinately to influence *plag-15* expression or related downstream pathways remains an open question for future investigation.

## Resource availability

### Lead contact

Further information and requests for resources and reagents should be directed to and will be fulfilled by the Lead Contact, Riekelt H. Houtkooper (r.h.houtkooper@amsterdamumc.nl).

### Materials availability

This study did not generate new materials.

### Data and code availability

• The lipidomics data is available via MetaboLights: MTBLS12324.
• The RNA-seq data is available via NCBI’s Sequence Read Archive under accession number PRJNA1234059.
• This study did not generate any codes.
• Any additional information is available from the lead contact upon request.

## Acknowledgments

We thank the *Caenorhabditis* Genetics Center (CGC) at the University of Minnesota for providing *C. elegans* strains. The CGC is funded by NIH Office of Research Infrastructure Programs (P40 OD010440). Work in the Houtkooper group is financially supported by the 10.13039/100007214Velux Stiftung (no. 1063) and an NWO-Middelgroot grant (no. 91118006) from 10.13039/501100003246Netherlands Organisation for Scientific Research. The project was supported by a Longevity Impetus Grant from Norn Group (to G.E.J. and R.H.H.). A.W.G. is supported by an Amsterdam UMC Postdoc Career Bridging Grant, a grant from the 10.13039/501100007601European Union’s Horizon 2020 research and innovation program under Marie Skłodowska-Curie grant agreement (no. 101108082), and AGEM Talent and Development grant. G.E.J. is supported by a VENI grant from 10.13039/501100001826ZonMw (no. 09150161810014). A.T. is supported by an NWO-FAPESP grant on healthy aging (no. 457002002). S.v.d.R. is supported by an 10.13039/501100003180AMC PhD scholarship.

## Author contributions

S.v.d.R., M.M., R.H.H., G.E.J., A.T., and A.W.G. designed the project. S.v.d.R., M.M., R.K., S.W.D., and W.L. performed experiments. S.v.d.R. and M.M. analyzed RNA-seq of the data. S.v.d.R., B.V.S., M.v.W., and F.M.V. performed and analyzed lipidomics. A.D.D. performed the bioinformatics analysis of the lipidomics data. S.v.d.R., R.H.H., G.E.J., A.T., and A.W.G. wrote the manuscript with the contribution of all the other authors.

## Declaration of interests

The authors declare no competing interests.

## STAR★Methods

### Key resources table

**Table:** 

REAGENT or RESOURCE	SOURCE	IDENTIFIER
**Bacterial and virus strains**		

*Escherichia coli*: OP50	*Caenorhabditis* Genetics Center	RRID:WB-STRAIN:OP50
*Escherichia coli*: HT115 (DE3)	*Caenorhabditis* Genetics Center	RRID:WB-STRAIN:HT115(DE3)
*plag-15* RNAi	Ahringer	*M05B5.4*
*hlh-30* RNAi	Ahringer	*W02C12.3*
*elt-3* RNAi	Ahringer	*K02B9.4*
*efl-1* RNAi	Ahringer	*Y102A5C.18*
*pmp-5* RNAi	Ahringer	*T10H9.5*
*mrp-4* RNAi	Ahringer	*F21G4.2*
*spin-3* RNAi	Ahringer	*F09A5.1*
*mfsd-8* RNAi	Ahringer	*Y53G8AR.7*
*vit-5* RNAi	Ahringer	*C04F6.1*
*vit-6* RNAi	Ahringer	*K07H8.6*
*abu-8* RNAi	Ahringer	*C03A7.14*

**Chemicals, peptides, and recombinant proteins**		

5-Fluorouracil (5-FU)	Sigma-Aldrich	Cat# F6627
Ampicillin sodium salt	Sigma-Aldrich	Cat# A9518
Carbenicillin disodium salt	Sigma-Aldrich	Cat# C1389
IPTG	AppliChem	Cat# A1008,0005
TRI reagent	Sigma-Aldrich	Cat# T3809
Lysotracker Red DND-99	Invitrogen	Cat# L7528
LysoSensor™ Green DND-189	Invitrogen	Cat# L7535
cOmplete™, Mini Protease Inhibitor Cocktail	Roche	Cat # 11836153001
RIPA buffer	Made in house	N/A
Pierce™ BCA Protein Assay Kits	Thermo Scientific	Cat# 23225
NuPAGE™ Bis-Tris Mini Protein Gels, 4–12%, 1.0 mm	Thermo Scientific	Cat# NP0321BOX
Methanol (Optima™ LC/MS Grade)	Fisher Chemical	Cat# A454SK-4
GFP antibody (FL): sc-8334	Santa Cruz	Cat# sc-8334; RRID: AB_641123
Alpha-Tubulin antibody	Sigma-Aldrich	Cat# T6199; RRID: AB_477583
IRDye 800 CW Goat anti-Rabbit IgG secondary antibody	LICORbio	Cat# 926-32211
IRDye 680 RD Donkey anti-Mouse IgG secondary antibody	LICORbio	Cat# 926-68072
18 MΩ MilliQ water	Made in house	N/A
Stainless metal bead (5 mm diameter)	Qiagen	Cat# 69989
Formic acid, Pierce™	Thermo Scientific	Cat# PI28905
2-Propanol	Merck	Cat# 34863
Chloroform	Merck	Cat# 1.02445
Methanol	Merck	Cat# 1.13351

**Critical commercial assays**		

QIAprep Spin Miniprep Kit	Qiagen	Cat #27104
RNase-Free DNase Set	Qiagen	Cat# 79254
RNeasy MinUlute Cleanup kit	Qiagen	Cat# 74204
QuantiTect Reverse Transcription Kit	Qiagen	Cat# 205314
LightCycler 480 SYBR Green I Master kit	Roche	Cat# 04887352001

**Deposited data**		

*C. elegans* lipidomics data	This paper	Table S3, Metabolights: MTBLS12324
*C. elegans* RNAseq data	This paper	Table S4, NCBI Sequence Read Archive: PRJNA1234059

**Experimental models: Organisms/strains**		

*C. elegans*: N2 Bristol	*Caenorhabditis* Genetics Center (CGC); https://cbs.umn.edu/cgc/home	RRID: WB-STRAIN: N2_(ancestral)
*C. elegans:* NL2099 *rrf-3(pk1426)*	*Caenorhabditis* Genetics Center (CGC); https://cbs.umn.edu/cgc/home	RRID: WB-STRAIN: NL2099
*C. elegans:* MAH235*: sqIs19 [hlh-30p::hlh30::gfp +rol-6(su1006)]*	*Caenorhabditis* Genetics Center (CGC); https://cbs.umn.edu/cgc/home	RRID: WB-STRAIN: MAH235
*C. elegans:* DA2123*: adIs2122 [lgg-1p::GFP::lgg-1 +rol-6(su1006)]*	*Caenorhabditis* Genetics Center (CGC); https://cbs.umn.edu/cgc/home	RRID: WB-STRAIN: DA2123

**Oligonucleotides**		

*cdc-42* Fw: AGTAATGATCGGTGGCGAGC	Sigma-Aldrich	N/A
*cdc-42* Rv: CCGTTGACACTGGTTTCTGC	Sigma-Aldrich	N/A
*F35G12.2* Fw: ACTGCGTTCATCCGTGCCGC	Sigma-Aldrich	N/A
*F35G12.2* Rv: TGCGGTCCTCGAGCTCCTTC	Sigma-Aldrich	N/A
*plag-15* Fw: GTGATTCTCGTGCCTGGTGA	Sigma-Aldrich	N/A
*plag-15* Rv: AATCGGCTGTCTGCTTGGAG	Sigma-Aldrich	N/A

**Software and algorithms**		

GraphPad Prism v9	GraphPad Software, Inc.	https://www.graphpad.com/scientificsoftware/prism/
R (version 4.2.2)	The R Foundation	https://www.r-project.org/
Adobe Illustrator 2023	Adobe	https://www.adobe.com/products/illustrator.html
Leica Application Suite X software	Leica Geosystems	https://www.leica-microsystems.com/products/microscope-software/p/leica-las-x-ls/
Openxlsx	openxlsx	https://cran.r-project.org/web/packages/openxlsx/index.html
Dplyr	Dplyr	https://CRAN.R-project.org/package=dplyr
Xlsx	xlsx	https://CRAN.R-project.org/package=xlsx
ggplot2	ggplot2	https://cran.r-project.org/web/packages/ggplot2/index.html
Pheatmap	pheatmap	https://cran.r-project.org/web/packages/pheatmap/
wrMTrck ImageJ plugin	ImageJ	https://www.phage.dk/plugins/wrmtrck.html
edgeR	Bioconductor	https://bioconductor.org/packages/release/bioc/html/edgeR.html
Limma/voom	Biocondutor	https://bioconductor.org/packages/release/bioc/html/limma.html

### Experimental model and study participant details

#### Worms strains and maintenance

*Caenorhabditis elegans* strains N2 Bristol and *rrf-3(pk1426)* were obtained from the *Caenorhabditis* Genetics Center (CGC; University of Minnesota, Minneapolis, MN, USA). Furthermore, *Escherichia coli (E. coli)* OP50 and DA2123: *adIs2122 [lgg-1p::GFP::lgg-1 + rol-6(su1006)]* were obtained from the CGC. MAH235: *sqIs19 [hlh-30p::hlh30::gfp + rol-6(su1006)]* was a kind gift from W.B. Mair (Harvard T.H. Chan School of Public Health, Harvard University, Boston, MA). Worms were maintained on nematode growth media (NGM; 2.5 g/L, Bacto-peptone, 3 g/L sodium chloride, 17 g/L select agar, 5mg/L cholesterol) at 20°C, as described previously.^55^

#### RNAi experiments

*E. coli* HT115 (DE3) with the empty vector (EV) L4440 was obtained from the CGC. Bacterial feeding RNAi experiments were performed as described before.^56^ RNAi bacteria used were *plag-15*, *pmp-5*, *hlh-30*, *elt-3*, *spin-3*, *mrp-4*, *mfsd-8*, and *efl-1* derived from the Ahringer library.^57^

### Method details

#### Lifespan analysis

At the L4 stage, worms were transferred from the maintenance plates to NGMi plates (NGM containing 2 mM IPTG, 25 mg/mL carbenicillin) seeded with different RNAi bacteria. Worms were allowed to develop into gravid adults overnight and removed after egg laying for 24 h. The worms were then raised until reaching the L4 stage and subsequently transferred to NGMi plates with RNAi bacteria containing 10 μM 5-Fluorouracil (5FU, Sigma Aldrich).^55^ A total of 100 worms were used per condition and were assessed every other day. Additionally, they were transferred to fresh NGMi plates on a weekly basis. After 10 days, worms were transferred from NGMi with 5FU to NGMi plates without 5FU. Worms that escaped, were missing, or exhibited symptoms such as an exploding vulva or internal egg hatching were excluded from the analysis. Statistical analysis was performed using the Log-rank (Mantel-Cox) tests on Kaplan-Meier curves with GraphPad PRISM 9.

#### Healthspan analysis – Mobility and body size

Synchronized worms were developed and grown until L4 on NGMi plates. Then they were transferred to NGMi supplemented with 5FU plates. Around 50 worms were transferred to clean NGM plates without seeding any RNAi bacteria. The worms were stimulated by tapping the plates 3 times and their movement was measured using a Leica (Amsterdam, The Netherlands) M205 FA fluorescent microscope for 200 cycles for 30 s in total. Images were captured using Leica Application Suite X software. After this, the images were processed using the wrMTrck plugin for ImageJ.^17^

For body size assays, worms were paralyzed by 40 μM tetramisole (Sigma Aldrich) and aligned on a clean NGM plate. They were imaged using the Leica and body size was measured and quantified via ImageJ.

#### Quantitative polymerase chain reaction (qPCR)

Worms were synchronized and ∼1000 harvested at day one and day six of adulthood. Worms were harvested as described before. TRIzol was added to the worms and they were lysed in the Tissuelyser II (QIAGEN) for 5 min at 30 times/sec. RNA was quantified with Nanodrop and 1 μg of extracted RNA was reverse transcribed into cDNA according to manufacturer’s instructions using the QuantiTect Reverse Transcription Kit (QIAGEN; Venlo, The Netherlands). Quantitative gene expression analysis was performed by using the LightCycler 480 SYBR Green I Master (Roche; Woerden, The Netherlands) and the quantitative gene expression was measured by the LightCycler 480 Instrument II (Roche). Primers were designed (Table S2) and the reference genes *cdc-42* and *f35g12.2* were used to measure *plag-15* expression.

#### Confocal microscopy

A synchronized *rrf-3* worm culture was treated from parental L4 worms with RNAi as described in the section ‘*worm strains and maintenance’* and around 50 worms were transferred to a new NGM plate when they reached either L4 or day 6 of adulthood. 300 μL lysotracker DND-99 (Molecular Probes) and lysosensor (Invitrogen) solution were added to the bacterial lawn of the plate. Worms were stained overnight and transferred to a new plate to recover for one hour. Approximately 50 worms were paralyzed in a drop of 40 μM tetramisole on an agarose pad and covered with a glass coverslip.^58^ Lysosomes of these worms were visualized by a Leica TCS SP8 SMD camera and the images were captured using Leica Application Suite X software. Worms were immobilized by 40 μM tetramisole and mounted on 2% agarose. They were visualized by Leica TCS SP8 SMD camera with a 40 × 1.30 oil CS2 objective lens. Images were captured with Leica Application Suite X.

Day 1 adult of *hlh-30* reporter worms were used to investigate the colocalization of HLH-30 to the nucleus, treated with EV or 50% *plag-15* RNAi from parental L4. Worms were washed with M9 buffer and incubated in 95% ethanol for 10 min. After another wash with M9 buffer, worms were stained with 1:500 DAPI and incubated for 10 min at room temperature. Afterward, worms were prepared on glass coverslips as described previously.

#### Western blotting

Worms were harvested and freeze-dried as described previously. Freeze-dried worm pellet was re-suspended in 300 μL RIPA buffer with cOmplete, Mini Protease Inhibitor Cocktail (Roche). Worm lysate was obtained by adding a 5 mm steel bead to the worm suspension and mixed using a TissueLyser II (Qiagen) for 2 × 2.5 min at frequency of 30 times/s, followed by tip sonication (energy level: 40 J; output: 8 W) for two times on ice-cold water. Protein quantification was performed with a BCA assay. *C. elegans* homogenates (30 μg) were subjected to electrophoresis on a NuPAGE Bis-Tris Mini Protein Gels, 4–12%, 1.0 mm and transferred to a nitrocellulose sheet. After blocking of non-specific binding sites with 3% BSA in phosphate buffered saline plus 0.1% (w/v) Tween-20 for 30 min, the blot was incubated overnight at 4°C with rabbit polyclonal antibodies raised against GFP Antibody (FL) (Santa Cruz, sc-8334) diluted 1:1000 in 0.5% BSA solution. As a loading control, the membranes were re-probed with a monoclonal antibody against tubulin (Sigma-Aldrich) using a 1:10000 dilution (incubation 2 h). Antigen-antibody complexes were visualized with IRDye 800CW goat anti-rabbit secondary antibody for GFP and IRDye 680RD donkey anti-mouse secondary antibody for tubulin using the Odyssey Infrared Imaging System (LI-COR Biosciences, Nebraska, USA).

#### RNA sequencing

N2 worms were synchronized, and treated with RNAi from the L1 stage. At the L4 stage, they were washed off the plates with M9 buffer followed by three times in water, before being snap-frozen in liquid nitrogen. Worms were freeze-dried and homogenized with Tissuelyser II (QIAGEN) for 5 min and 30 Hz in TRIzol (Invitrogen), and total RNA was isolated with TRI-zol reagent (Sigma-Aldrich). RNase-Free DNase (Qiagen) was used to remove genomic DNA and samples were cleaned with RNeasy MinUlute Cleanup kit (Qiagen). RNA concentration was determined with a NanoDrop 2000 spectrophotometer (Thermo Scientific, The Netherlands) and stored at −80°C.

RNA libraries were prepared and sequenced with the Ilumina platform by Genome Scan (Leiden, The Netherlands). To process the samples, we used the NEBNext Ultra II Directional RNA Library Prep Kit for Illumina. Preparation of the samples were performed according to the protocol "NEBNext Ultra II Directional RNA Library Prep Kit for Illumina" (NEB #E7760S/L). mRNA was isolated from total RNA by using oligo-dT magnetic beads. After the mRNA was fragmented, cDNA synthesis was performed. This was used for ligation with the sequencing adapters and PCR amplification of the resulting product. To determine the quality and yield of the samples, Fragment Analyzer was used. The size of the resulting products was consistent with the expected size distribution (a broad peak between 300 and 500 bp). Clustering and DNA sequencing using the NovaSeq6000 were performed according to manufacturer’s protocols. A concentration of 1.1 nM of DNA was used. NovaSeq control software NCS v1.6 was used.

Reads were subjected to quality control FastQC^59^ trimmed using Trimmomatic v0.32^60^ and aligned to the *C. elegans* genome obtained from Ensembl (wbcel235.v91), using HISAT2 v2.1.0.^61^ Counts were obtained using HTSeq (v0.11.0, default parameters)^62^ using the corresponding GTF considering the directions of the reads. Statistical analyses were performed using the edgeR v3.26.8^63^ and limma/voom v 3.40.6^64^ R packages. All genes with more than 2 counts in at least 3 of the samples were kept. Count data were transformed to log2-counts per million (logCPM), which was normalized by applying the trimmed mean of M-values method^63^ and precision weighted using voom.^65^ Differential expression was assessed using an empirical Bayes moderated t-test within limma’s linear model framework including the precision weights estimated by voom.^64^^,^^65^ Resulting *p* values were corrected for multiple testing using the Benjamini-Hochberg false discovery rate. Data processing was performed using R v3.6.1 and Bioconductor v3.9. Genes were re-annotated using biomaRt using the Ensembl genome databases (v91). Functional annotation clustering of differentially expressed genes was performed using The Database for Annotation, Visualization and Integrated Discovery (DAVID) bioinformatics resource with all default settings.^66^ Unprocessed RNAseq data is available at NCBI’s Sequence Read Archive under accession number PRJNA1234059. Processed RNAseq data is available in Table S4.

#### Lipidomics

Worms were treated from the parental L4 stage and harvested on L4 and day 6 of adulthood. Worm pellets were homogenized, and the lipids were extracted as described before.^67^ Lipidomics was performed as previously described, with minor adjustments.^68^^,^^69^ Briefly: In a 2 mL tube, containing ∼4000 worms, the following amounts of internal standard dissolved in 1:1 (v/v) methanol:chloroform were added for lipidomics: Bis(monoacylglycero)phosphate BMP(14:0)^2^ (0.2 nmol), ceramide-1-phosphate C1P (d18:1/12:0) (0.125 nmol), D_7_-cholesteryl ester CE(16:0) (2.5 nmol), ceramide Cer(d18:1/12:0) (0.125 nmol), ceramide Cer(d18:1/25:0) (0.125 nmol), cardiolipin CL(14:0)^4^ (0.1 nmol), diacylglycerol DAG(14:0)^2^ (0.5 nmol), glucose ceramide GlcCer(d18:1/12:0) (0.125 nmol), lactose ceramide LacCer(d18:1/12:0) (0.125 nmol), lysophosphatidic acid LPA(14:0) (0.1 nmol), lysophosphatidylglycerol LPG(14:0) (0.02 nmol), phosphatidic acid PA(14:0)^2^ (0.5 nmol), phosphatidylcholine PC(14:0)^2^ (2 nmol), phosphatidylethanolamine PE(14:0)^2^ (0.5 nmol), phosphatidylglycerol PG(14:0)^2^ (0.1 nmol), phosphatidylinositol PI(8:0)^2^ (0.5 nmol), phosphatidylserine PS(14:0)^2^ (5 nmol), sphinganine 1-phosphate S1P(d17:0) (0.125 nmol), sphinganine-1-phosphate S1P(d17:1) (0.125 nmol), ceramide phosphocholines SM(d18:1/12:0) (2.125 nmol), sphingosine SPH(d17:0) (0.125 nmol), sphingosine SPH(d17:1) (0.125 nmol), triacylglycerol TAG(14:0)^2^ (0.5 nmol). Subsequently, solvents were added to achieve a total volume of 500 μL water, 500 μL methanol and 1 mL chloroform. *C. elegans* were homogenized before the addition of chloroform using a Qiagen TissueLyser II for 5 min at 30 times/second with a 5 mm Qiagen Stainless Steel Bead in each tube. All samples were thoroughly mixed, before centrifugation for 10 min at 14.000 rpm.

The bottom layer, containing the apolar phase, was transferred to a clean 1.5 mL tube and evaporated under a stream of nitrogen at 60°C. The residue was dissolved in 100 μL of 1:1 (v/v) methanol:chloroform. Lipids were analyzed using a Thermo Scientific Ultimate 3000 binary HPLC coupled to a Q Exactive Plus Orbitrap mass spectrometer. For normal phase separation, 2 μL of each sample was injected onto a Phenomenex LUNA silica, 250 ∗ 2 mm, 5μm 100 Å. Column temperature was held at 25°C. Mobile phase consisted of (A) 85:15 (v/v) methanol:water containing 0.0125% formic acid and 3.35 mmol/L ammonia and (B) 97:3 (v/v) chloroform:methanol containing 0.0125% formic acid. Using a flow rate of 0.3 mL/min, the LC gradient consisted of: dwell at 10% A 0–1 min, ramp to 20% A at 4 min, ramp to 85% A at 12 min, ramp to 100% A at 12.1 min, dwell at 100% A 12.1–14 min, ramp to 10% A at 14.1 min, dwell at 10% A for 14.1–15 min. For reversed phase separation, 5 μL of each sample was injected onto a Waters HSS T3 column (150 × 2.1 mm, 1.8 μm particle size). Column temperature was held at 60°C. Mobile phase consisted of (A) 4:6 (v/v) methanol:water and B 1:9 (v/v) methanol:isopropanol, both containing 0.1% formic acid and 10 mmol/L ammonia. Using a flow rate of 0.4 mL/min, the LC gradient consisted of: dwell at 100% A at 0 min, ramp to 80% A at 1 min, ramp to 0% A at 16 min, dwell at 0% A for 16–20 min, ramp to 100% A at 20.1 min, dwell at 100% A for 20.1–21 min. MS data were acquired using negative and positive ionization using continuous scanning over the range of m/z 150 to m/z 2000. Quality control measurements were performed at the start of the run, after 12 samples and after the final sample. Data were analyzed using an in-house developed lipidomics pipeline written in the R programming language (http://ww.r-project.org) and MATLAB, which has been successfully applied in several peer-reviewed publications.^70^^,^^71^ The data analysis pipeline is available upon request. For lipid classes detected in multiple measurement modes only one mode is selected based on maximum separation and sensitivity. All reported lipids were normalized to corresponding internal standards according to lipid class, as well as to freeze-dried tissue weight. Lipid identification has been based on a combination of accurate mass (maximum deviation 5 parts per million), (relative) retention times, fragmentation spectra, analysis of samples with known metabolic defects, and the injection of relevant standards, as described previously.^72^ The notation “-O” indicates lipids that contain an alkyl-ether group, “P” indicates an alkenyl-ether group. When lipids are labeled with both the “O” and “P” (“O+P”), this indicates that it is unknown what the ether species is, or it could not be separated chromatographically. The lipidomics data have been deposited to MetaboLights[1] repository with the study identifier MTBLS12324.^73^

### Quantification and statistical analysis

Comparisons between more than two groups were performed by using One-way ANOVA test, while comparisons with two groups, a student t-test was used. The log-rank test was used for lifespan analysis. Prism 9 software was used for statistical analysis. P-values below 0.05 were significant (∗∗∗∗*p* < 0.0001; ∗∗∗*p* < 0.001; ∗∗*p* < 0.01; ∗*p* < 0.05; ns, not significant).

## Supplemental information

Document S1. Figures S1–S6, Tables S1, and S2

Table S3. *C. elegans* lipidomics data of EV and *plag-15* RNAi worms, related to Figure 2

Table S4. *C. elegans* RNA-seq data of EV and *plag-15* RNAi worms at L4 stage, related to Figure 3
