# Supplementary material for: Targeting phospholipase PLAG-15 promotes healthy aging in C. elegans via lysosomal-related genes
Source: iScience. 2025 Jun 16;28(7):112880. doi: 10.1016/j.isci.2025.112880 (PMC12246578; doi:10.1016/j.isci.2025.112880)
Supplement: Document S1. Figures S1–S6, Tables S1, and S2 [file mmc1.pdf]

iScience, Volume *28*

Supplemental information

Targeting phospholipase PLAG-15 promotes healthy aging in *C. elegans* via lysosomal-related genes

Sanne van der Rijt, Marte Molenaars, Rashmi Kamble, Weisha Li, Bauke V. Schomakers, Adrie D. Dane, Simone W. Denis, Michel van Weeghel, Frédéric M. Vaz, Alessandra Tammaro, Georges E. Janssens, Arwen W. Gao, and Riekelt H. Houtkooper

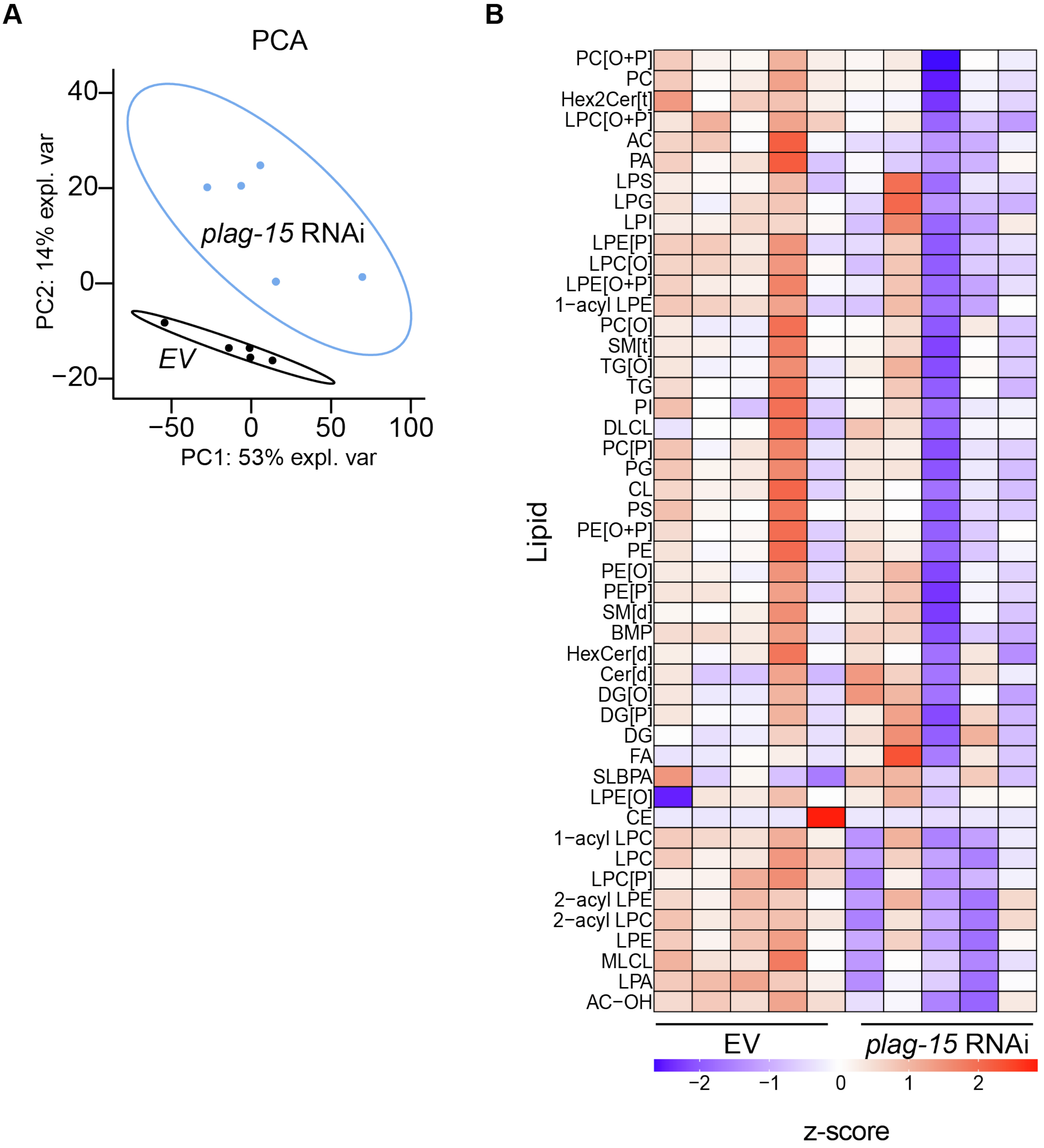

Figure S1. Worms fed with *plag-15* RNAi display a distinct lipidomic profile compared with control worms. (**A**) Principal Component Analysis (PCA) plot of the lipidome showing group separation based on the lipidome in *plag-15* RNAi versus empty vector (EV) control. (**B**) Heatmap of the full lipidome of *plag-15* RNAi worms compared to EV. N=5 per group.

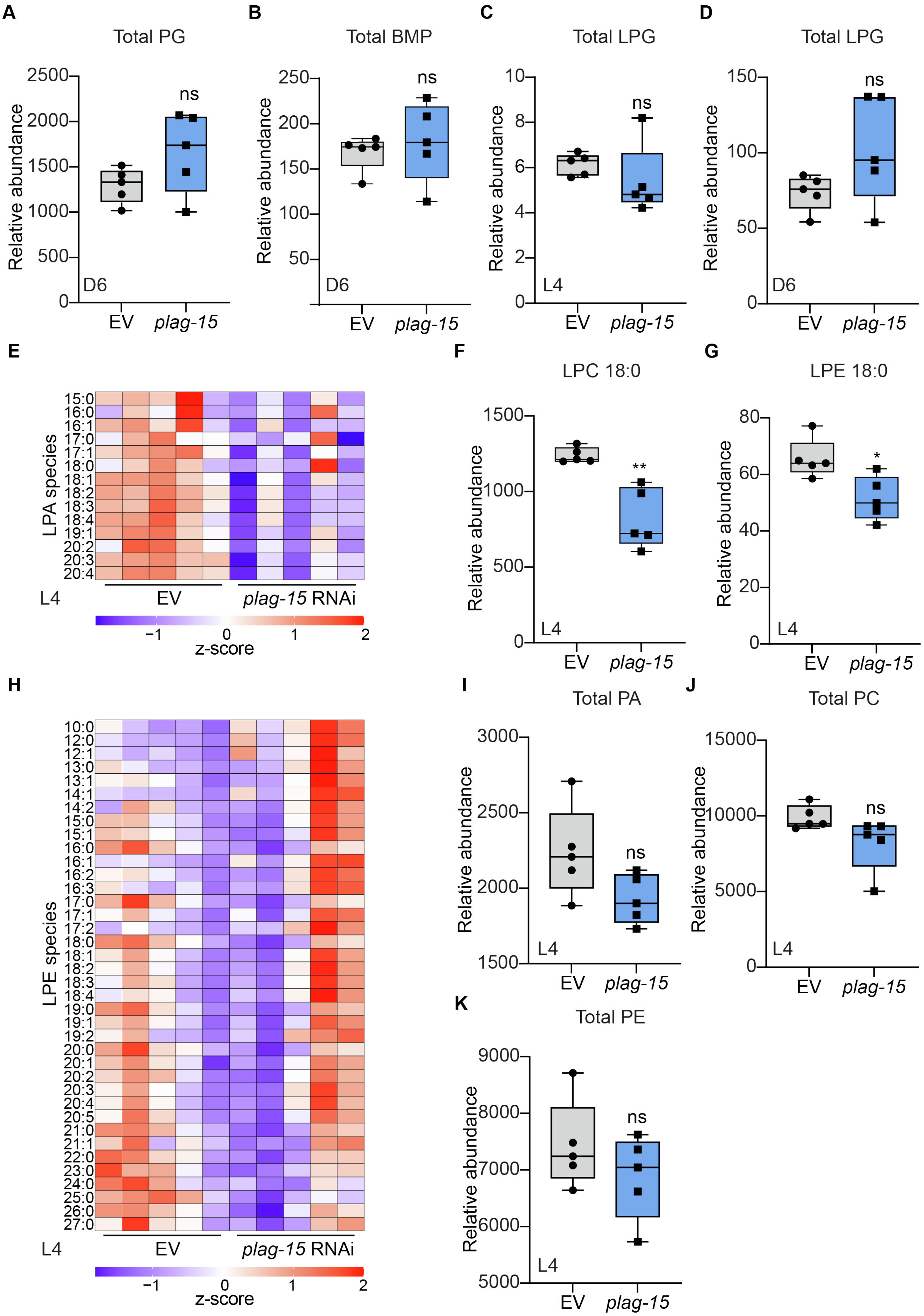

Figure S2. Lipidomic changes in *plag-15* RNAi compared to control. (**A**) Total PG abundance in day 6 of adulthood worms, in empty vector (EV) control and *plag-15* RNAi worms. (**B**) Total Bis(monoacylglycero)phosphate (BMP) abundance in Day 6 of adult worms fed with EV and to *plag-15* RNAi. (**C-D**) Total lysophosphatidylglycerol (LPG) abundance in L4

(**C**) and Day 6 adult (**D**) worms fed with control and *plag-15* RNAi. (**E**) Heatmap of lysophosphatidic acid (LPA) lipid species in EV and *plag-15* RNAi worms collected at L4 stage. (**F-G**) Lipid abundance of lysophosphatidylcholine (LPC) 18:0 (**F**) and lysophosphatidylethanolamine (LPE) 18:0 (**G**) in *plag-15* RNAi worms compared to EV worms. (**H**) Heatmap of lysophosphatidic acid (LPA) lipid species in EV and *plag-15* RNAi worms collected at L4 stage. (**I-K**) Lipid abundance of total phosphatidic acid (PA) (**I**), phosphatidylcholine (PC) (**J**), phosphatidylethanolamine (PE) (**K**) *plag-15* RNAi worms compared to EV worms at the L4 stage. N=5 per group. Data are presented as mean +/- SD.

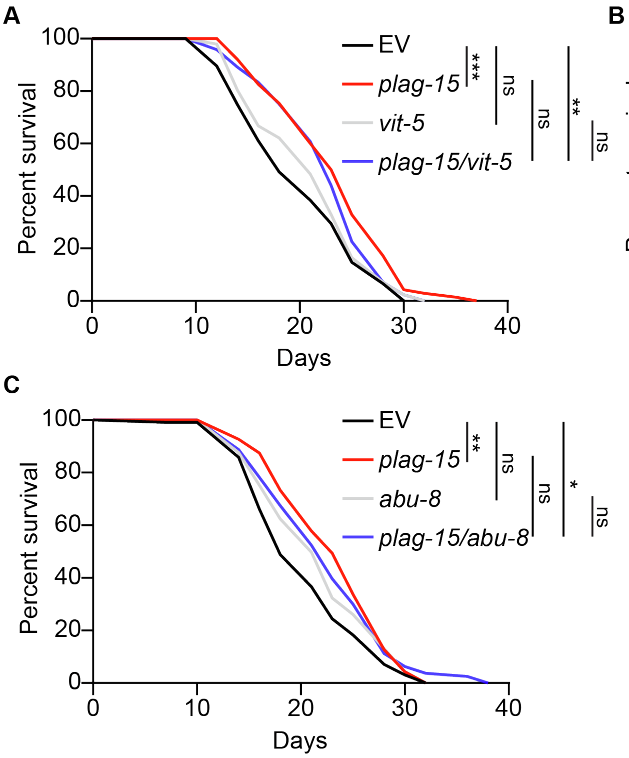

Figure S3. Lifespan validations of genes related to defense response and unfolded protein response. (**A-C**) Lifespan curves of the defense response and unfolded protein response genes of the GO terms. (**A**) Lifespan curve of EV, *plag-15*, *vit-5* and *plag-15/vit-5* RNAi. Lifespan extension of *plag-15* RNAi is not dependent on *vit-5.* (**B**) Lifespan curve of EV, *plag-15*, *vit-6* and *plag-15/vit-6* RNAi. Lifespan extension of *plag-15* RNAi is dependent on *vit-6.* (**C**) Lifespan curve of EV, *plag-15*, *abu-8* and *plag-15/abu-8* RNAi*.* Lifespan extension of *plag-15* RNAi is not dependent on *abu-8***.** Statistical comparison was determined by using the log-rank test. *p <0.05; **p <0.01, ***p <0.001, and ****p <0.0001. See Table S1 for lifespan statistics.

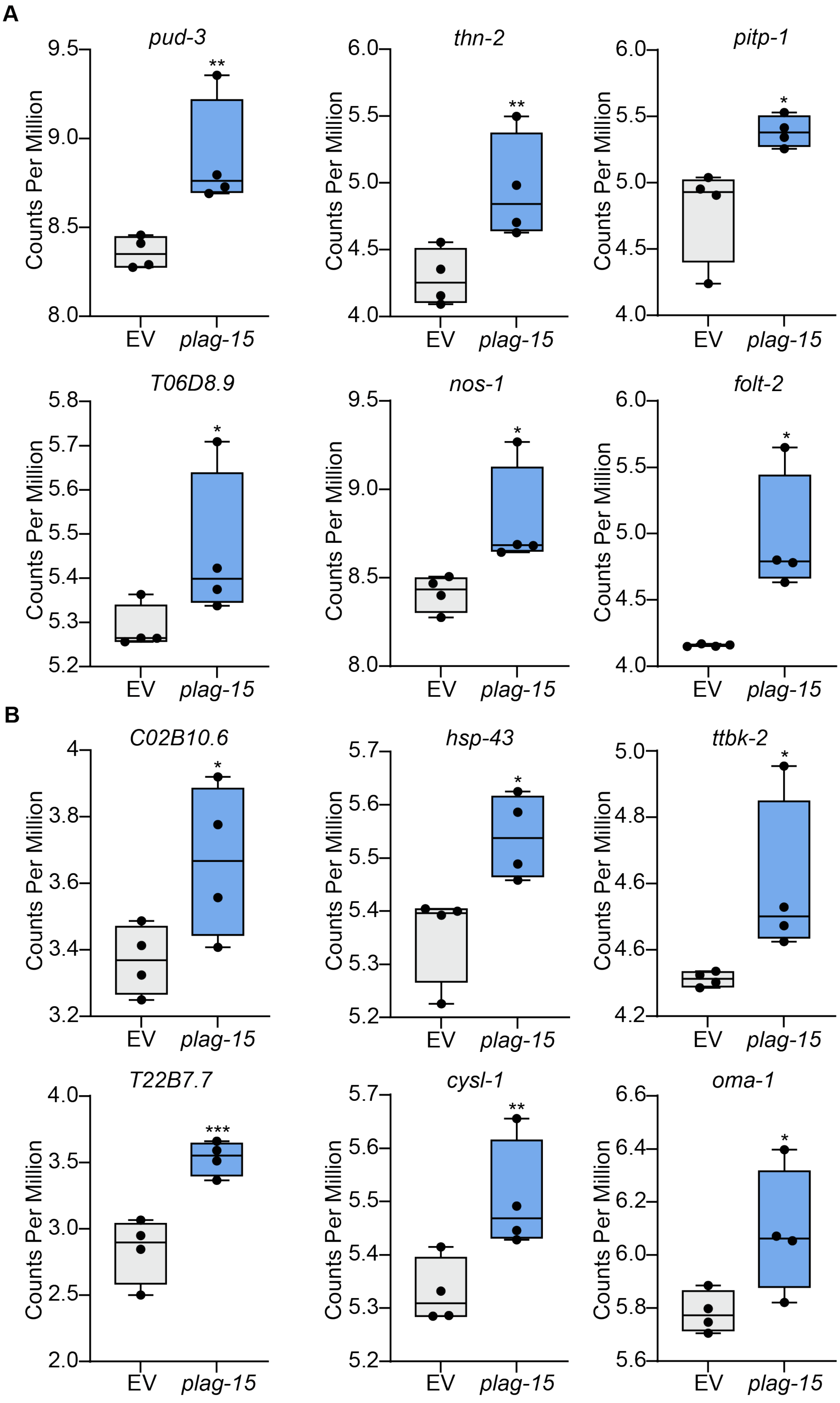
A
pud-3
thn-2
pitp-1
T06D8.9
nos-1
folt-2
B
C02B10.6
hsp-43
ttbk-2
T22B7.7
cysl-1
oma-1
Counts Per Million
EV
plag-15
*
**
***

Figure S4. Upregulated genes targeted by transcription factor *elt-3* or *hlh-30* (**A**) Genes targeted by *elt-3*. (**B**) Genes targeted by *hlh-30.* *: p <0.05 **: p < 0.01: *** < 0.001. n=4 per group. Data are presented as mean +/- SD.

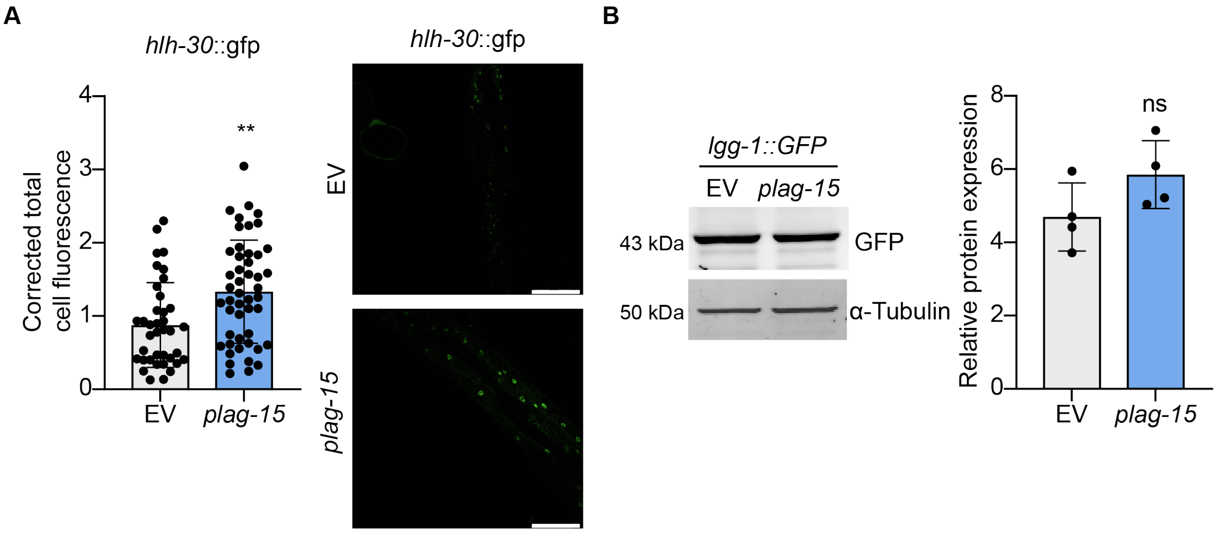

Figure S5. *hlh-30*::GFP fluorescent intensity of *plag-15* RNAi worms. (**A**) Corrected total fluorescent intensity and confocal images of *hlh-30*::GFP worms fed with EV (n=38) and *plag-15* RNAi (n=48). *plag-15* RNAi worms show increased *hlh-30* translocation to the nucleus. Scale bar = 50 µm. Statistical analysis was performed by t-test. (**B**) Western blot of *lgg-1::GFP* worms treated with EV (n=4) and *plag-15* (n=4). No statistical difference was found in the levels of LGG-1 (p=0.1280). Statistical analysis was performed by t-test. Data are presented as mean +/- SD.

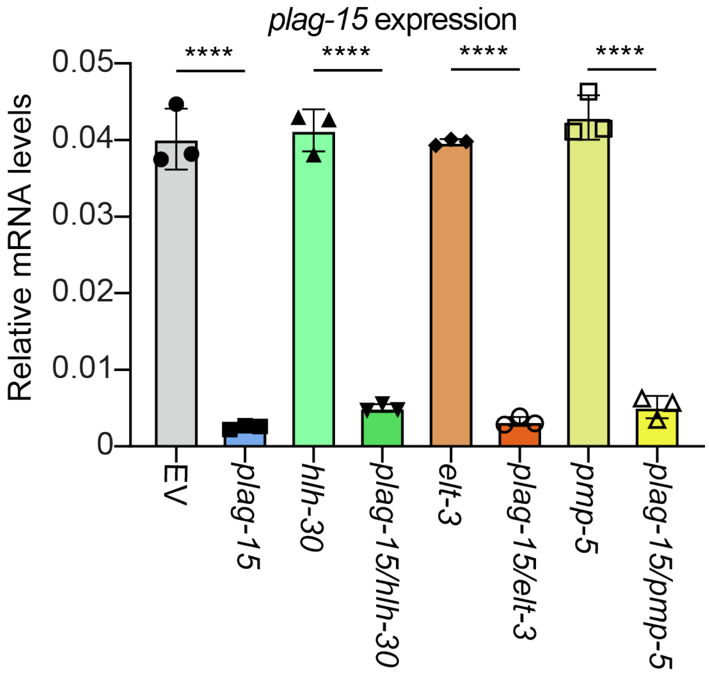

Figure S6. *plag-15* knockdown efficiency upon double knockdown in L4 worms. The mRNA levels of *plag-15* in EV, *plag-15, hlh-30, plag-15/hlh-30, elt-3, elt-3/plag-15, pmp-5, pmp-5/hlh-30* RNAi worms (compared to reference genes *cdc-42* and *F35G12.2)*. Knockdown efficiency of *plag-15* alone is similar to *plag-15/hlh-30, plag-15/elt-3* and *plag-15/pmp-5* double RNAi. Statistical comparison was determined by using One-way ANOVA: ****p <0.0001. See Table S2 for qPCR primers used. N=3 per group. Data are presented as mean +/- SD.

Table S1. Lifespan statistics.

	Treatment	Median lifespan (days)	% change	Number animals (died/total)	P-value against control group
Figure 3 panel A					
Replicate 1*	EV	21		78/99	
	plag-15	23	9.5%	78/106	0.0014
	hlh-30	18	-14.3%	98/103	0.0053
	hlh-30/plag-15	18	-14.3%	83/101	0.0171
	hlh-30 vs hlh-30/plag-15				0.7149
Replicate 2	EV	21		76/99	
	plag-15	25	19%	58/85	0.0234
	hlh-30	19	-9.5%	94/98	<0.0001
	hlh-30/plag-15	19	-9.5%	95/97	0.0013
	hlh-30 vs hlh-30/plag-15				0.0905
Figure 3 panel B					
Replicate 1*	EV	21		78/99	
	plag-15	23	9.5%	78/106	0.0014
	elt-3	21	0%	69/101	0.4847
	elt-3/plag-15	21	0%	90/105	0.4887
	elt-3 vs elt-3/plag-15				0.9762
Replicate 2	EV	18		84/95	
	plag-15	22	22.2%	79/104	<0.0001
	elt-3	18	0%	83/105	0.7396
	elt-3/plag-15	18	0%	71/108	0.6713
	elt-3 vs elt-3/plag-15				0.9252
Figure 3 panel C					
Replicate 1*	EV	19		77/97	
	plag-15	27	42.1%	66/90	0.0011
	efl-1	17	-10.5%	67/93	0.0477
	efl-1/plag-15	22	15.8%	79/95	0.3970
	efl-1 vs efl-1/plag-15				0.0058
Replicate 2	EV	18		84/95	
	plag-15	22	22.2%	79/104	<0.0001
	efl-1	18	0%	74/94	0.0639
	efl-1/plag-15	18	0%	66/78	0.2631
	efl-1 vs				0.0027

	efl-1/plag-15				
Figure 3 panel D					
Replicate 1*	EV	21		78/99	
	plag-15	23	9.5%	78/106	0.0014
	pmp-5	23	9.5%	76/95	0.0188
	pmp-5/plag-15	21	0%	102/110	0.5024
	pmp-5 vs pmp-5/plag-15				0.0724
Replicate 2	EV	21		76/99	
	plag-15	25	19.0%	58/85	0.0234
	pmp-5	21	0%	85/102	0.4334
	pmp-5/plag-15	19	-9,5%	71/98	0.6698
	pmp-5 vs pmp-5/plag-15				
Replicate 3	EV	18		84/95	
	plag-15	22	22.2%	79/104	<0.0001
	pmp-5	20	11.1%	83/99	0.0043
	pmp-5/plag-15	18	0%	94/103	0.5667
	pmp-5 vs pmp-5/plag-15				0.0334
Figure 3 panel E					
Replicate 1*	EV	19		77/97	
	plag-15	27	42.1%	64/90	0.0011
	spin-3	19	0%	71/98	0.4681
	spin-3/plag-15	22	15.8%	80/97	0.0142
	spin-3 vs spin-3/plag-15				0.0974
Replicate 2	EV	18		84/95	
	plag-15	22	22.2%	79/104	<0.0001
	spin-3	18	0%	53/91	0.9990
	spin-3/plag-15	20	11.1%	69/103	0.197
	spin-3 vs spin-3/plag-15				0.0358
Figure 3 panel F					
Replicate 1*	EV	19		77/97	
	plag-15	27	42.1%	64/90	0.0011
	mrp-4	19	0%	78/97	0.6781
	mrp-4/plag-15	22	15.8%	72/91	0.0183
	mrp-4 vs mrp-4/plag-15				0.0336
Replicate 2	EV	18		84/95	

	plag-15	22	22.2%	79/104	<0.0001
	mrp-4	18	0%	83/96	0.5490
	mrp-4/plag-15	18	0%	88/100	0.0856
	mrp-4 vs mrp-4/plag-15				0.0218
Figure 3 panel F					
Replicate 1*	EV	19		77/97	
	plag-15	27	42.1%	64/90	0.0011
	mfsd-8	22	15.7%	68/87	0.0175
	mfsd-8/plag-15	27	42.1%	85/96	<0.0001
	mfsd-8 vs mfsd-8/plag-15				0.2266
Replicate 2	EV	18		84/95	
	plag-15	22	22.2%	79/104	<0.0001
	mfsd-8	18	0%	73/101	0.2305
	mfsd-8/plag-15	20	11.1%	84/97	0.0009
	mfsd-8 vs mfsd-8/plag-15				0.0245
Figure S5 panel A					
Replicate 1*	EV	18		85/107	
	plag-15	25	38.8%	75/96	0.0002
	vit-5	21	16.6%	87/100	0.2213
	vit-5/plag-15	23	27.7%	85/108	0.0057
	vit-5 vs vit-5 /plag-15				0.1441
Replicate 2	EV	21		80/103	
	plag-15	25	19%	47/101	0.0003
	vit-5	23	9.5%	62/99	0.6033
	vit-5/plag-15	25	19%	63/102	0.0038
	vit-5 vs vit-5 /plag-15				0.0175
Figure S5 panel B					
Replicate 1*	EV	18		85/107	
	plag-15	25	38.8%	75/96	0.0002
	vit-6	18	0%	91/98	0.0543
	vit-6/plag-15	18	0%	94/104	0.5917
	vit-6 vs vit-6 /plag-15				0.1564
Replicate 2	EV	21		80/103	
	plag-15	25	19%	47/101	0.0003
	vit-6	18	-14.3%	93/100	0.0016

	vit-6/plag-15	18	-14.3%	85/101	0.0098
	vit-6 vs vit-6 /plag-15				0.5803
Figure S5 panel C					
Replicate 1*	EV	18		100/110	
	plag-15	23	27.7%	72/100	0.0027
	abu-8	21	16.6%	79/101	0.0767
	abu-8/plag-15	23	27.7%	88/104	0.0107
	abu-8 vs abu-8/plag-15				0.4394
Replicate 2	EV	18		93/105	
	plag-15	21	16.6%	95/102	0.0015
	abu-8	21	16.6%	102/96	0.0005
	abu-8/plag-15	21	16.6%	97/100	0.01
	abu-8 vs abu-8/plag-15				0.2353

* experiment shown in the figures

Table S2: Primers used for *C. elegans* qPCR

**Gene**	**Forward (sequence 5'→3')**	**Reverse (sequence 5'→3')**
**Reference gene**		
cdc-42	AGTAATGATCGGTGGCGAGC	CCGTTGACACTGGTTTCTGC
F35G12.2	ACTGCGTTCATCCGTGCCGC	TGCGGTCCTCGAGCTCCTTC
**Target gene**		
plag-15	GTGATTCTCGTGCCTGGTGA	AATCGGCTGTCTGCTTGGAG
